# Prostaglandin E2 inhibits Tr1 cell differentiation through suppression of c-Maf

**DOI:** 10.1371/journal.pone.0179184

**Published:** 2017-06-12

**Authors:** Kirsten Mary Hooper, Weimin Kong, Doina Ganea

**Affiliations:** Department of Microbiology and Immunology, Lewis Katz School of Medicine, Temple University, Philadelphia, Pennsylvania, United States of America; Purdue University, UNITED STATES

## Abstract

Prostaglandin E2 (PGE2), a major lipid mediator abundant at inflammatory sites, acts as a proinflammatory agent in models of inflammatory/autoimmune diseases by promoting CD4 Th1/Th17 differentiation. Regulatory T cells, including the IL-10 producing Tr1 cells counterbalance the proinflammatory activity of effector Th1/Th17 cells. Tr1 cell differentiation and function are induced by IL-27, and depend primarily on sustained expression of c-Maf in addition to AhR and Blimp-1. In agreement with the in vivo proinflammatory role of PGE2, here we report for the first time that PGE2 inhibits IL-27-induced differentiation and IL-10 production of murine CD4^+^CD49b^+^LAG-3^+^Foxp3^-^ Tr1 cells. The inhibitory effect of PGE2 was mediated through EP4 receptors and induction of cAMP, leading to a significant reduction in c-Maf expression. Although PGE2 reduced IL-21 production in differentiating Tr1 cells, its inhibitory effect on Tr1 differentiation and c-Maf expression also occurred independent of IL-21 signaling. PGE2 did not affect STAT1/3 activation, AhR expression and only marginally reduced Egr-2/Blimp-1 expression. The effect of PGE2 on CD4^+^CD49b^+^LAG-3^+^ Tr1 differentiation was not associated with either induction of Foxp3 or IL-17 production, suggesting a lack of transdifferentiation into Foxp3^+^ Treg or effector Th17 cells. We recently reported that PGE2 inhibits the expression and production of IL-27 from activated conventional dendritic cells (cDC) in vivo and in vitro. The present study indicates that PGE2 also reduces murine Tr1 differentiation and function directly by acting on IL-27-differentiating Tr1 cells. Together, the ability of PGE2 to inhibit IL-27 production by cDC, and the direct inhibitory effect on Tr1 differentiation mediated through reduction in c-Maf expression, represent a new mechanistic perspective for the proinflammatory activity of PGE2.

## Introduction

The proinflammatory activity of effector T cells is counterbalanced by regulatory T cell subsets, *i*.*e*. the Foxp3+ regulatory T cells (Treg) and the IL-10 producing regulatory type 1 cells (Tr1). Both thymus-derived tTreg and peripherally-induced pTreg are characterized by constitutive expression of CD25 and of the master transcription factor Foxp3, and are essential to maintain self-tolerance [1, 2]. Tr1 cells, generated in the periphery, secrete high levels of IL-10 and co-express LAG-3 and CD49b without constitutive expression of Foxp3 and CD25 [3, 4]. Tr1 cells mediate immune tolerance in organ transplantation, alleviate and prevent disease progression in inflammatory bowel disease (IBD) models, and are currently tested in clinical trials for IBD and graft-versus host disease following hematopoietic stem cell transplantation [3, 5–8].

IL-27, a p28/EBI3 heterodimer produced by activated antigen-presenting cells (APCs), promotes Tr1 differentiation and function. The immunosuppressive role of IL-27 has been described in various inflammatory disease models, with IL-27R deficient mice exhibiting excessive inflammation, and IL-27 treatment inducing IL-10 and suppressing IL-17 production [9, 10]. In vitro, IL-27 promotes the differentiation of immunosuppressive, IL-10 producing Tr1 cells [11–14]. In vivo, mice deficient in IL-27R show significant reduction in Tr1 cells during infection [15]. In contrast, transgenic mice constitutively expressing GITR ligand in APCs have higher levels of IL-27 and Tr1 cells [16]. Tr1 cells were also generated in vivo following intranasal administration of anti-CD3 in a process dependent on IL-27 production by upper airway resident DC [17].

In mice, IL-27 induction of Tr1 differentiation occurs through STAT1 and STAT3 activation followed by induction and/or activation of c-Maf, AhR, and Egr-2/Blimp-1 [18, 19]. AhR and c-Maf complexes activate the transcription of both *Il10* and *Il21* genes, with IL-21 and IL-10 subsequently serving as growth/stabilizer factors for Tr1 [14, 20, 21]. An additional AhR/c-Maf-independent pathway leading to *Il10* expression involves activation of the STAT3-dependent Egr-2/Blimp-1 pathway [22]. The role of these different signaling molecules was confirmed in studies using conditional knockouts, where deficient Tr1 differentiation and/or IL-10 production was observed in c-Maf^-/-^, Blimp-1^-/-^, IL-21R^-/-^, and AhR mutant T cells [14, 21, 23–25].

In contrast to substantial information on Tr1 induction, only extracellular ATP and hypoxia were reported recently to inhibit Tr1 differentiation [26]. To our knowledge, there are no reports on the role of lipid mediators such as prostaglandins on IL27-induced Tr1 differentiation.

Prostaglandin E2 (PGE2), the most abundant cyclooxygenase product generated at inflammatory sites, signals through four EP receptors (EP1-4) that vary in affinity, signal duration and signaling pathways [27, 28]. In vitro, the effects of PGE2 on CD4 T cell differentiation depend on the use of purified T cells or of co-cultures with conventional dendritic cells (cDCs). In the presence of cDCs, PGE2 inhibits Th1 and promotes Th17 differentiation indirectly through inhibition of IL-12 and upregulation of IL-23 production in cDC [29–31]. However, when TCR-stimulated CD4 T cells are differentiated in polarizing conditions in the absence of APCs, PGE2 promotes both Th1 and Th17 differentiation primarily through upregulation of cytokine receptors [32–34]. In vivo, PGE2 acts as a proinflammatory mediator in models of contact hypersensitivity, IBD, rheumatoid arthritis (RA), and experimental autoimmune encephalomyelitis (EAE). This effect is mostly associated with increases in Th1/Th17 differentiation and/or function [32, 35–38].

The effect of PGE2 on the differentiation and function of regulatory T cells is less studied. PGE2 action on Foxp3^+^ Treg is still under debate with reports of both increases and decreases in Foxp3 expression and Treg function [39, 40]. To our knowledge, there are no reports at the present time on the role of PGE2 in IL-27 induced Tr1 differentiation. We have recently reported that PGE2 reduces in vitro and in vivo IL-27 production in TLR-stimulated cDC, which could subsequently impact Tr1 differentiation [41]. Here we report on the direct effect of PGE2 on Tr1 differentiation of TCR-stimulated naïve CD4 T cells cultured in the presence of IL-27. In our experimental system, PGE2 reduced the percentage of CD4^+^CD49b^+^LAG-3^+^Foxp3^-^ T cells and IL-10 production within the CD4^+^CD49b^+^LAG-3^+^ Tr1 population. The inhibitory effect was mediated through the EP4 receptor and cAMP and associated with significant reduction of c-Maf expression.

## Materials and methods

### Mice

C57BL/6 (6–10 weeks old) and B6(Cg)-*Il10*^*tm1*.*1Karp*^ (*Il10*^*gfp*^) mice were purchased from Jackson Laboratory. 129S6/SvEv-*Stat1*^*tm1Rds*^ (*Stat1*^*-/-*^) and wild type (WT) mice (129S6/SvEv) were purchased from Taconic Farms. Euthanasia was performed using exposure to carbon dioxide followed by cervical dislocation. Mice were housed and maintained in accordance with protocol #4317 approved by IACUC at Temple University.

### Reagents

Prostaglandin E2, LPS (*Escherichia coli* O55:B5), recombinant mouse IFNγ and DNase were purchased from Sigma-Aldrich. Recombinant mouse IL-27p28, recombinant mouse IL-21, recombinant mouse IL-17A and neutralizing anti-mouse IL-27 antibodies were from R&D Systems. Recombinant TGF-β1 was from Peprotech. Butaprost, misoprostol, sulprostone, dimethyl-PGE2, the specific activator of the exchange protein activated by cAMP (EPAC) 8-pCPT-2′-O-Me-cAMP (8-pCPT), the PI3K inhibitor LY294002 and EP receptor antagonists PF-04418948 and ONOAE3-208 were purchased from Cayman Chemical. Dibutyryl-cAMP was from Calbiochem. Recombinant antibodies anti-CD3 (Armenian Hamster monoclonal IgG, clone 145-2C11), anti-CD28 (Syrian Hamster monoclonal IgG, clone 37.51), and capture and biotinylated anti-mouse IL-17A were purchased from BioLegend. Capture and biotinylated anti-mouse antibodies for IL-21 and IL-27 ELISA were from R&D Systems. Streptavidin-HRP was purchased from BioLegend. Tetramethylbenzidine substrate reagent set was from BD Biosciences. Anti-mouse CD49b PE antibody and isotype control were from BioLegend. APC-conjugated anti-mouse LAG-3, anti-mouse LAG-3 PE-Cy7, anti-mouse CD4 PerCP-Cy5.5, FoxP3 PerCP-Cy5.5, Egr-2 APC, c-Maf eFluor660, FITC pSTAT3 and isotype control antibodies were purchased from eBioscience. FITC conjugated anti-mouse CD4 and AlexaFluor 647-conjugated anti-mouse Blimp-1 was from BD Biosciences. Phospho-STAT1 (Tyr701) PE (clone 58D6) and isotype control were purchased from Cell Signaling Technology.

### T cell isolation and culture

Naïve CD4^+^CD62L^+^ T cells were isolated from spleens of 6–8 week old mice using a T cell isolation MicroBead kit per manufacturer’s instructions (Miltenyi Biotec). Purified, naïve T cells were cultured in vitro in fresh RPMI supplemented with 10% heat-inactivated FBS, 2 mM L-glutamine, 1% antibiotics and 50 μM βME. Cells were stimulated with plate-bound anti-CD3 (3 μg/ml) and soluble anti-CD28 (1 μg/ml) in the presence of recombinant IL-27 (50 ng/ml) for three days to derive Tr1 cells. Recombinant IL-2 (10 ng/ml) was added to all cultures on day 2.

Alternatively, *Il10*^*gfp*^ mice were injected intraperitoneally with anti-CD3 (20 μg/mouse) or vehicle (PBS) in addition to either dmPGE2 (200μg/kg) or vehicle (0.4% DMSO in PBS) twice, 48 hours apart. Four hours later, mice were euthanized and Tr1 cell populations within the Peyer’s patches, spleens and small intestines were analyzed. Briefly, Peyer’s patches were removed from the small intestine, and tissue was dissociated in the presence of 0.5 mg/ml liberase TL (Roche Diagnostics) and DNase to obtain single cell suspensions. Intraepithelial lymphocytes (IEL) and lamina propria lymphocytes (LPL) were isolated from the small intestine as previously described [42]. Samples were analyzed by FACS for Tr1 cell markers.

### FACS analysis

Cells were washed twice in FACS buffer (2mM EDTA, 0.5% BSA in PBS), incubated at 4^°^C for 30 minutes with anti-mouse CD4, CD49b, LAG-3, or corresponding isotype controls then washed three times. Cells were then either collected immediately using BD FACSCanto or underwent further staining for transcription factors. For intracellular staining, cells were fixed and permeabilized using BD Cytofix/Cytoperm per manufacturer’s instructions, incubated at 4^°^C for 30 minutes with anti-mouse FoxP3, c-Maf, Blimp-1, Egr-2 or isotype control. Alternatively, cells were fixed and permeabilized using eBioscience IC Fixation buffer per manufacturer’s instructions (eBioscience Protocol C) then stained at 4^°^C for 30 minutes with anti-mouse pSTAT3 or pSTAT1 antibody. Cells were washed three times then collected using BD FACSCanto. For all experiments, stained cell preparations were compared to isotype controls in order to identify and gate on positive cell populations. Data was analyzed using FlowJo (Ashland, OR) and GraphPad Prism 5.0.

### qRT-PCR

Gene expression analysis of *Maf*, *Ahr*, and *β-actin* was performed using SYBR-green based qRT-PCR. The following primer sets were used: *Ahr* sense 5’-CCACCCCTGCTGACAGAAAT-3’ and antisense 5’-AGCCATTCAGCGCCTGTAAC-3’, *Maf* sense 5’-AGCAGTTGGTGACCATGTCG-3’ and antisense 5’-TGGAGATCTCCTGCTTGAGG-3’, *β-actin* sense 5’-AGCTTCTTTGCAGCTCCTTCGTTGC-3’ and antisense 5’-ACCAGCGCAGCGATATCG TCA-3’. Expression levels of each gene were calculated using relative standard curves and normalized to *β-actin*.

### Cytokine ELISA

Cytokine levels in cell culture supernatants were quantified by sandwich ELISA. Splenocytes were plated in 24-well culture plates (2 x 10^6^/ml) and stimulated for 72 hours. Alternatively, purified T cells were plated in 24-well culture plates (1 x 10^6^/ml) and stimulated for 72 hours. Detection limit was 5 pg/ml for IL-27, 62.5 pg/ml for IL-21, and 15.6 pg/ml for IL-17.

### Statistics

Results represent mean ± SD. Statistical significance between groups was determined using unpaired t-test or one way ANOVA followed by Bonferroni correction (*p <0.05, **p <0.01, and ***p <0.001). Graphs were generated and statistical analysis performed using GraphPad Prism 5.0.

## Results

### PGE2 inhibits Tr1 differentiation of TCR-stimulated CD4 T cells

IL-27 functions as an essential factor in the differentiation of CD4^+^IL-10^+^Foxp3^-^ Tr1 cells [11, 12, 21, 43]. Recently, co-expression of CD49b and LAG-3 was identified as a characteristic of Tr1 cells [4]. We confirmed that IL-27 induces differentiation of naïve CD4+ T cells into CD49b^+^LAG-3^+^Foxp3^-^ Tr1 cells, in contrast to TGFβ1 which induces Foxp3^+^ Treg (S1 Fig). In cells treated with IL-27, PGE2 reduced the percentage of Tr1 cells, without inducing Foxp3 expression (S1 Fig).

**Fig 1:**
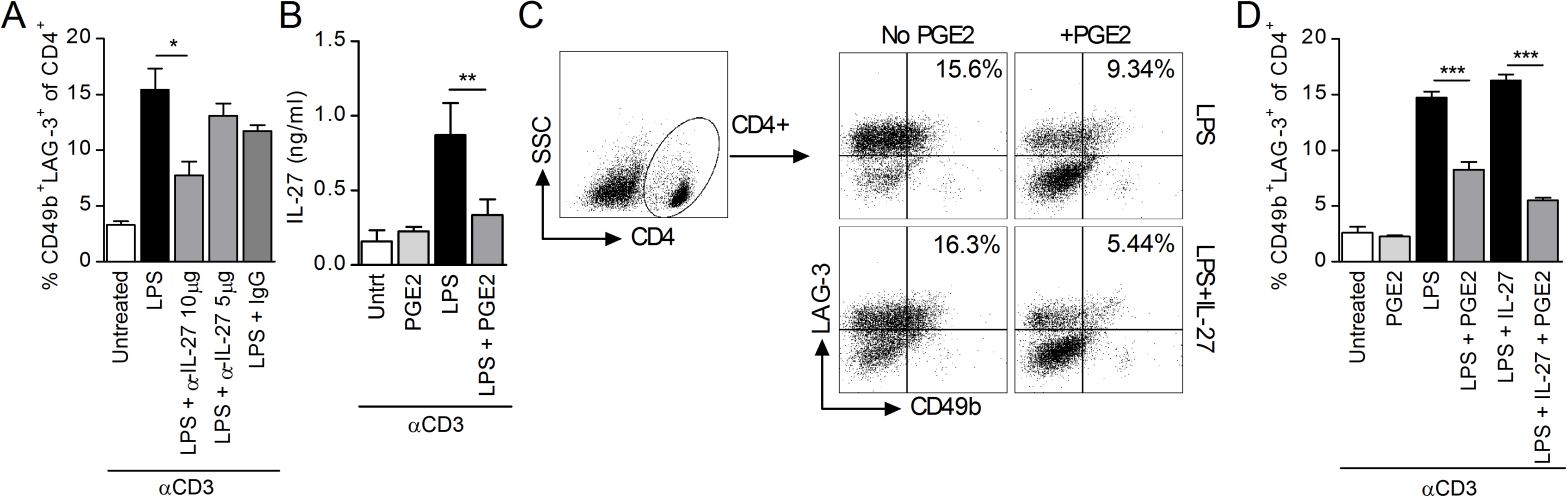
PGE2 inhibits Tr1 cell differentiation in splenocyte cultures. (A) *il10*^*gfp*^ splenocytes were stimulated with 3 μg/ml anti-CD3 and 1 μg LPS in the presence of neutralizing IL-27 antibodies (5 and 10 μg/ml) or IgG control (20 μg/ml). Cells were collected on day 3 and CD4^+^CD49b^+^LAG-3^+^ Tr1 cells were identified by FACS. Data are cumulative from two independent experiments. (B) Splenocytes were stimulated with anti-CD3 and LPS in the presence of PGE2 (10^-6^M). Supernatant was collected on day 3 and IL-27 levels analyzed by ELISA. (C-D) Splenocytes were stimulated with anti-CD3 and LPS in the presence or absence of IL-27 (50 ng/ml) and PGE2 (10^-6^M). Cells were collected on day 3 and CD4^+^CD49b^+^LAG-3^+^ Tr1 cells were identified by FACS. (C) Representative sample shows gating strategy to determine percentage of CD49b^+^LAG-3^+^ within CD4^+^ T cells and (D) graph presents representative data from three independent experiments. Each sample was tested in duplicate and results represent means ± SD. Significance was evaluated by one-way ANOVA; **P<0.*01*.

Next we confirmed the role of endogenous IL-27 in Tr1 differentiation by measuring the percentage of CD4^+^CD49^+^LAG-3^+^ cells in spleen cell cultures treated with LPS and anti-CD3 in the presence and absence of neutralizing anti-IL-27 antibodies. As expected, neutralization of endogenous IL-27 resulted in a decrease in CD4^+^CD49b^+^LAG-3^+^ cells (Fig 1A). Recently, we reported that PGE2 inhibits IL-27 production in TLR-stimulated macrophages and cDC [41]. We observed a similar effect in unfractionated spleen cells treated with LPS/anti-CD3 and PGE2 (Fig 1B). To determine whether PGE2 affects Tr1 differentiation solely through the reduction in endogenous IL-27, we tested its effect in the presence and absence of exogenously added IL-27 (concentrations 50 fold higher than the amount of endogenous IL-27 determined by ELISA). As expected, PGE2 reduced the percentage of CD4^+^CD49b^+^LAG-3^+^ cells. However, we observed similar levels of inhibition in the presence and absence of exogenous IL-27 (Fig 1C and 1D), suggesting that PGE2 also inhibits Tr1 differentiation independent of its effect on IL-27 production.

**Fig 2:**
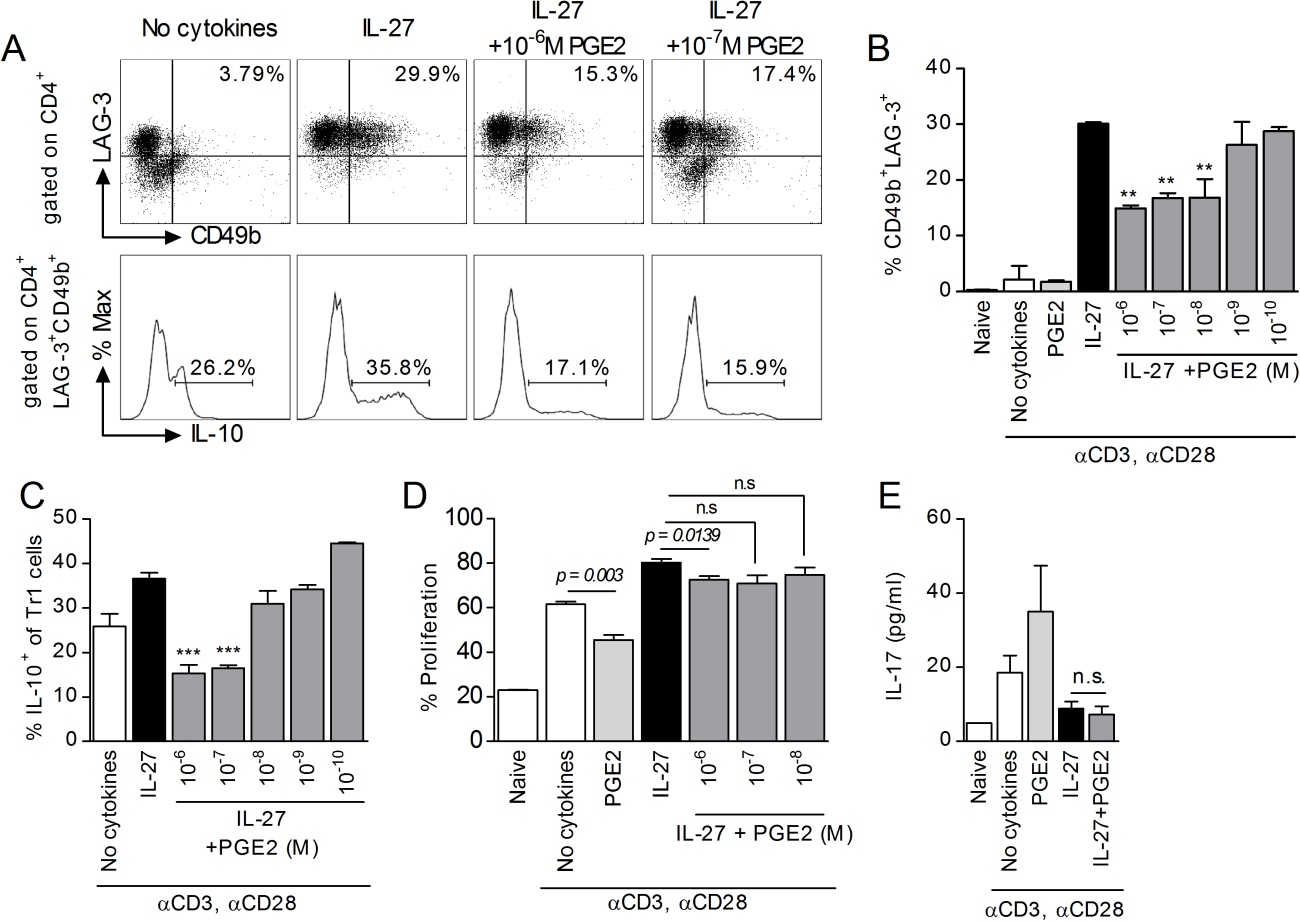
PGE2 inhibits IL-27 induced differentiation of Tr1 cells. (A-C) Naïve CD4^+^CD62L^+^ cells from *il10*^*gfp*^ mice were stimulated with plate-bound anti-CD3 (3 μg/ml) and soluble anti-CD28 (1 μg/ml) in the presence of 50 ng/ml IL-27 and various concentrations of PGE2 for three days. Cells were collected on day 3 and analyzed by FACS for Tr1 markers, and IL-10 within CD49b^+^LAG-3^+^ populations. Representative samples show CD49b^+^LAG-3^+^ populations (upper panel) and histograms of IL-10 (lower panel). Data are representative of four independent experiments. Each sample was tested in duplicate and results represent means ± SD. Significance was determined using one-way ANOVA and * represents the P value for a sample versus IL-27 control. **P<0.*01*, ***P<0.*001*. (D) For cell proliferation experiments, naïve CD4^+^CD62L^+^ cells were stained with CFSE per manufacturer’s instructions prior to stimulation with anti-CD3, anti-CD28, IL-27 and PGE2. Incorporation of CFSE was analyzed by FACS on day 3. Data are representative of two independent experiments. Each sample was tested in triplicate and results represent means ± SD. Significance was determined using unpaired t-test. (E) Naïve CD4^+^CD62L^+^ cells were stimulated in the presence of 50 ng/ml IL-27 and 10^-6^M PGE2. Supernatant was collected on day 3 and subjected to ELISA to determine IL-17 levels. Data are representative of three independent experiments. Each sample was tested in duplicate and results represent means ± SD. Significance was determined using one-way ANOVA.

To characterize the possible direct effect of PGE2 on T cells, we induced Tr1 differentiation in purified naïve CD4 T cells in polarizing conditions (anti-CD3/anti-CD28/IL-27). PGE2 reduced the percentages of CD4^+^CD49b^+^LAG-3^+^ T cells in a dose-dependent manner (Fig 2A and 2B), as well as the percentage of IL-10 expressing cells within the CD4^+^CD49^+^LAG-3^+^ population (Fig 2C). The inhibitory effect could be due to a general reduction in T cell proliferation. Indeed, PGE2 reduced proliferation in Th0 cells (anti-CD3/anti-CD28 stimulated in the absence of exogenous cytokines). However, PGE2 did not substantially affect proliferation in Tr1 polarizing conditions, at concentrations that reduced the percentage of CD4^+^CD49b^+^LAG-3^+^ Tr1 cells (Fig 2D). Therefore, it is unlikely that the inhibitory effect of PGE2 on the percentage of Tr1 cells and on IL-10 production within the Tr1 population is a result of a reduction in T cell proliferation.

Recently Th17 cells were reported to transdifferentiate in vivo into IL-10 producing Tr1 cells during resolution of inflammation or in conditions favoring IL-27 at the expense of IL-23 signaling [24, 44]. It is not known however whether a reciprocal transdifferentiation from Tr1 into Th17 cells can occur in vivo or in vitro. Since we and others reported that PGE2 contributes to Th17 differentiation/function by increasing IL-23 production in cDC and upregulating IL-23R expression in CD4 T cells [30, 32, 33, 45], we investigated whether PGE2 can induce IL-17 production in Tr1 polarizing conditions. In the absence of IL-27, PGE2 increased IL-17 production from activated CD4 T cells. In contrast, the levels of IL-17 secreted by CD4 T cells in Tr1 polarizing conditions were significantly lower, and they were not altered by PGE2 (Fig 2E). This suggests that inhibition of Tr1 differentiation by PGE2 is not associated with transdifferentiation to the Th17 phenotype.

### The inhibitory effect of PGE2 on Tr1 differentiation is mediated through EP4 and cAMP

**Fig 3:**
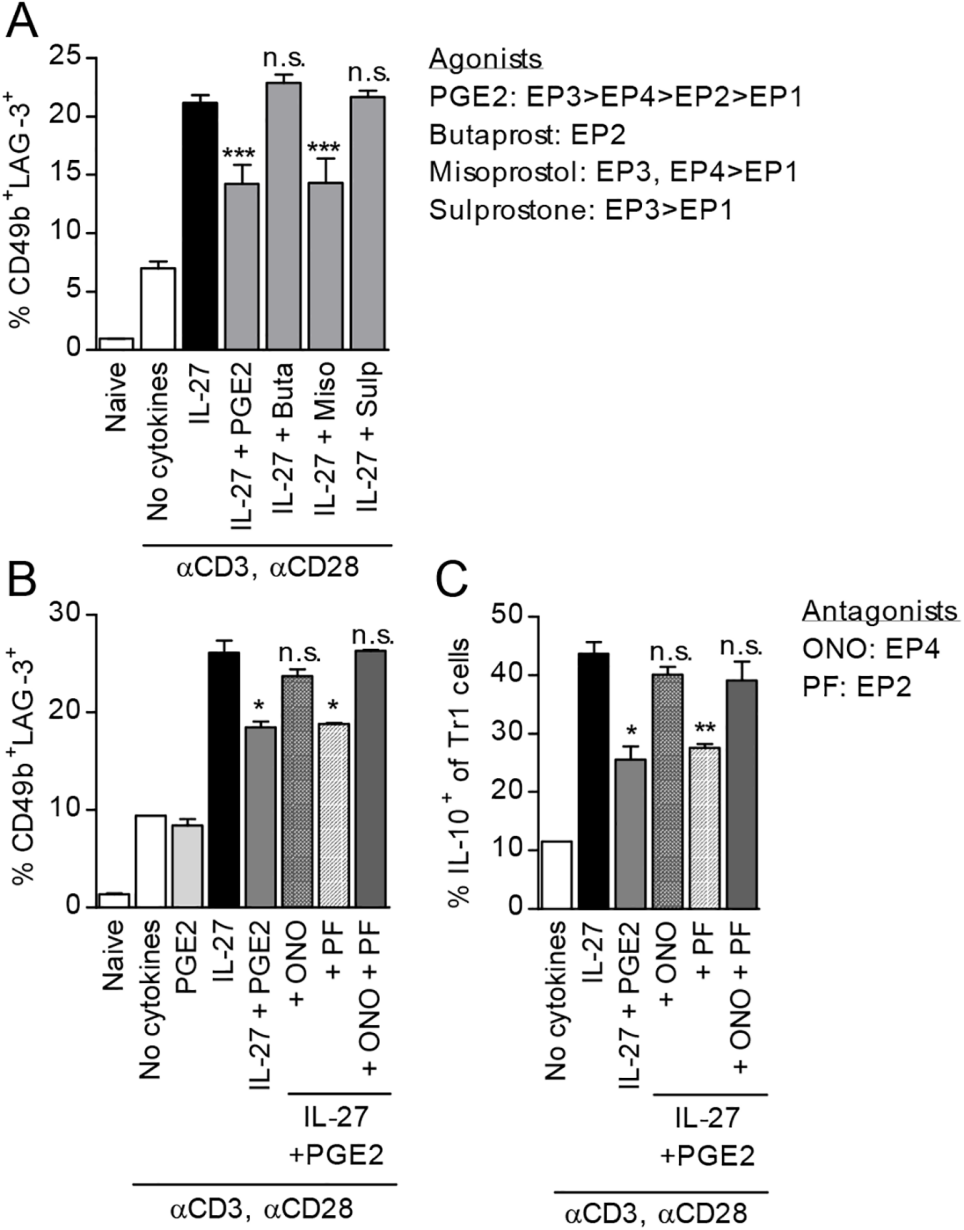
PGE2 signals through EP4 to inhibit Tr1 cell differentiation and expression of IL-10. (A) Naïve CD4^+^CD62L^+^ cells were stimulated in the presence of IL-27 and 10^-6^M PGE2 or 10^-5^M of EP receptor agonists: Butaprost (EP2), Misoprostol (EP3, EP4>EP1) or Sulprostone (EP3>EP1). Cells were collected on day 3 and analyzed by FACS for Tr1 cell markers. (B,C) Naïve CD4^+^CD62L^+^ cells from *il10*^*gfp*^ mice were pretreated for 30 minutes with 10^-6^M EP receptor antagonists, ONO-AE3-208 (ONO; EP4) or PF-04418948 (PF; EP2) prior to treatment with 50 ng/ml IL-27 and 10^-7^M PGE2. Cells were collected on day 3 and analyzed by FACS for Tr1 markers and IL-10 expression. Data are representative of three independent experiments. Each sample was tested in duplicate and results represent means ± SD. Significance was evaluated by one-way ANOVA and * symbolizes significance of experimental condition versus IL-27 control; *P<0.*05*, **P<0.*01*, ***P<0.*001*.

Immune cells, including activated CD4 T cells preferentially express the PGE2 receptors EP2 and EP4 [34, 46]. EP4 was shown to be the major contributor to Th1 and Th17 differentiation and expansion in vitro and in vivo in several autoimmune/inflammatory models [32, 34, 47]. To investigate which receptors are involved in the effects of PGE2 on Tr1 differentiation, we differentiated naïve CD4 T cells in the presence of IL-27 and various EP receptor agonists. Misoprostol (EP3, EP4>EP1) mimicked the effects of PGE2, while neither Butaprost (EP2) nor Sulprostone (EP3>EP1) altered Tr1 cell populations (Fig 3A). These results suggest that PGE2 signals through EP4 to inhibit Tr1 cell differentiation. We confirmed the involvement of EP4 by using the EP4 antagonist ONO-AE3-208 which reversed the inhibitory effect of PGE2 on both CD4^+^CD49^+^LAG-3^+^ Tr1 differentiation and on the percentage of IL-10+ Tr1 cells. In contrast, the EP2 antagonist PF-04418948 did not have any effect (Fig 3B and 3C). Although the agonist studies cannot exclude a potential synergistic effect of EP3 and EP4, the high levels of EP4 expression as compared to EP3 in CD4 T cells [48, 49], the effect of the specific EP4 antagonist, and the involvement of cAMP (see below), all point to EP4 as the major receptor involved in the PGE2 inhibition of Tr1 differentiation.

**Fig 4:**
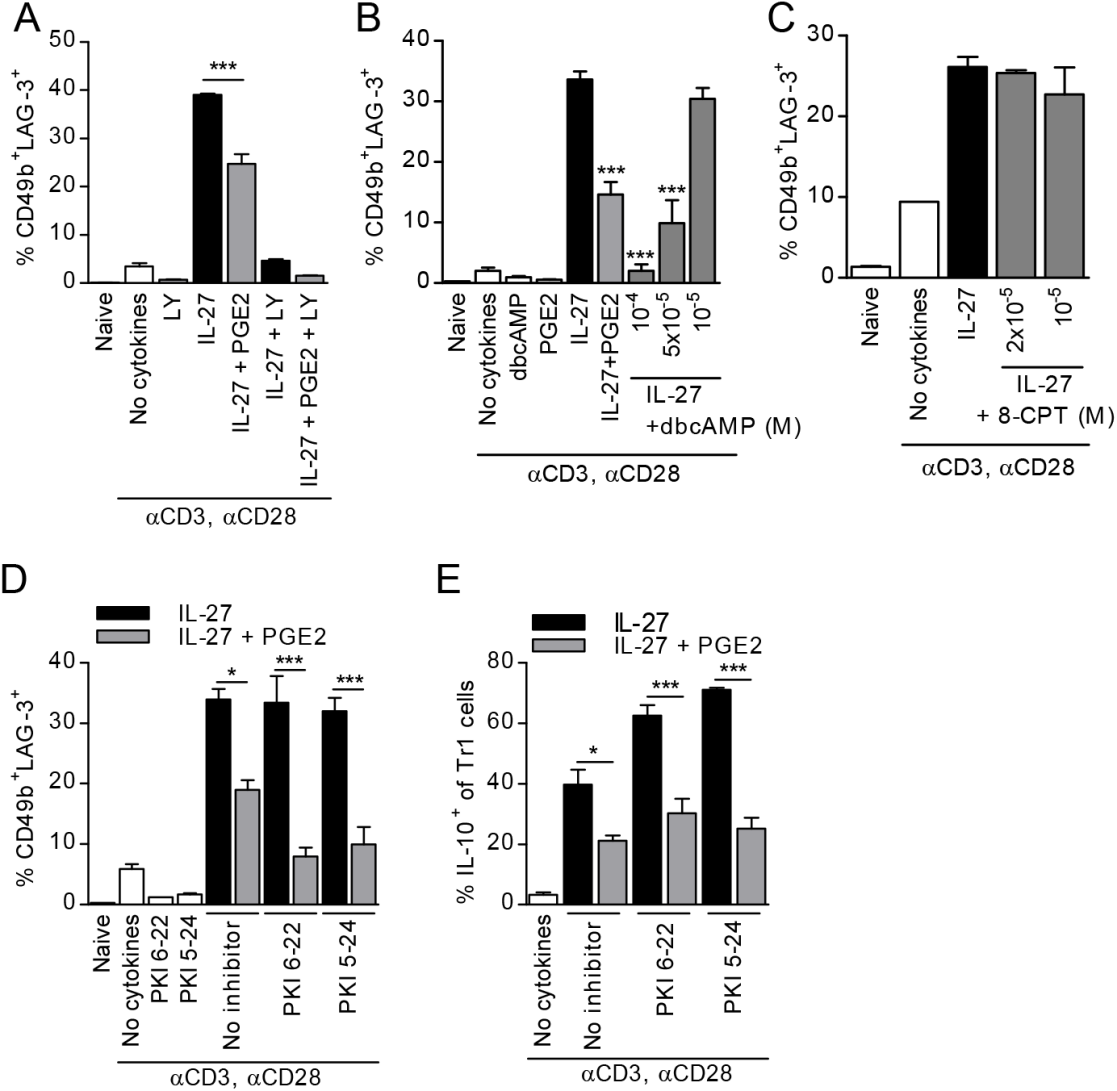
PGE2 signals through cAMP, but not EPAC or PKA, to inhibit IL-27 mediated Tr1 differentiation. (A) *il10*^*gfp*^ naïve CD4^+^CD62L^+^ cells were pretreated with PI3K inhibitor LY294002 (LY) for 30 min prior to stimulation in the presence of IL-27 and PGE2. (B) Naïve CD4^+^CD62L^+^ cells were stimulated as described in the presence of dbcAMP. (C) Cells were stimulated in the presence of a cell-permeable EPAC activator 8-pCPT-2′-O-Me-cAMP (8-CPT). Cells were collected on day 3 and analyzed by FACS for Tr1 markers. (D, E) Naïve CD4^+^CD62L^+^ cells were pretreated with PKA inhibitors PKI 6–22 and PKI 5–24 (2x10^-5^M) for 30 minutes prior to stimulation with IL-27 and PGE2 and analyzed on day 3 for Tr1 markers (D) and IL-10 within Tr1 cell populations (E). Data are representative of three independent experiments. Each sample was tested in duplicate and results represent means ± SD. Significance was evaluated by one-way ANOVA and * symbolizes significance of experimental condition versus IL-27 control (B); *P<0.*05*, **P<0.*01*, ***P<0.*001*.

EP4 signaling includes both generation of cAMP and activation of PI3K [50]. In agreement with previous reports showing that activation of PI3K is essential for T cell proliferation and survival [51–53], the PI3K inhibitor LY294002 proved to be detrimental for the generation of Tr1 cells in the presence as well as absence of PGE2 (Fig 4A). To investigate the potential involvement of cAMP in the inhibitory effect of PGE2, we used the cell-permeable cAMP analog dibutyryl-cAMP (dbcAMP). At high concentrations (10^-4^M) dbcAMP affected T cell viability. However, 5x10^-5^M dbcAMP, which did not affect T cell viability, reduced Tr1 cell differentiation similar to PGE2 (Fig 4B). We next investigated the role of exchange protein activated by cAMP (EPAC) by utilizing the specific EPAC activator 8-pCPT-2′-O-Me-cAMP (8-CpT) and found that 8-CpT did not reduce Tr1 cell differentiation (Fig 4C). To determine whether PKA activation is involved, we pretreated naïve CD4 T cells with two PKA inhibitor peptides, PKI 6–22 and PKI 5–24 prior to treatment with IL-27 and PGE2. Neither PKI 6–22 nor PKI 5–24 affected Tr1 differentiation in the absence of PGE2, or reversed the inhibitory effect of PGE2 (Fig 4D). Although inhibition of the PKA pathway actually increased the percentage of IL-10^+^ cells within the Tr1 cell population in the absence of PGE2, it did not reverse the inhibitory effect of PGE2 on the percentage of CD49b^+^LAG-3^+^IL-10^+^ T cells (Fig 4E). These results suggest that the inhibitory effect of PGE2 on Tr1 differentiation is dependent on cAMP through a signaling pathway independent of EPAC or PKA activation.

### PGE2 inhibits expression of c-Maf

**Fig 5:**
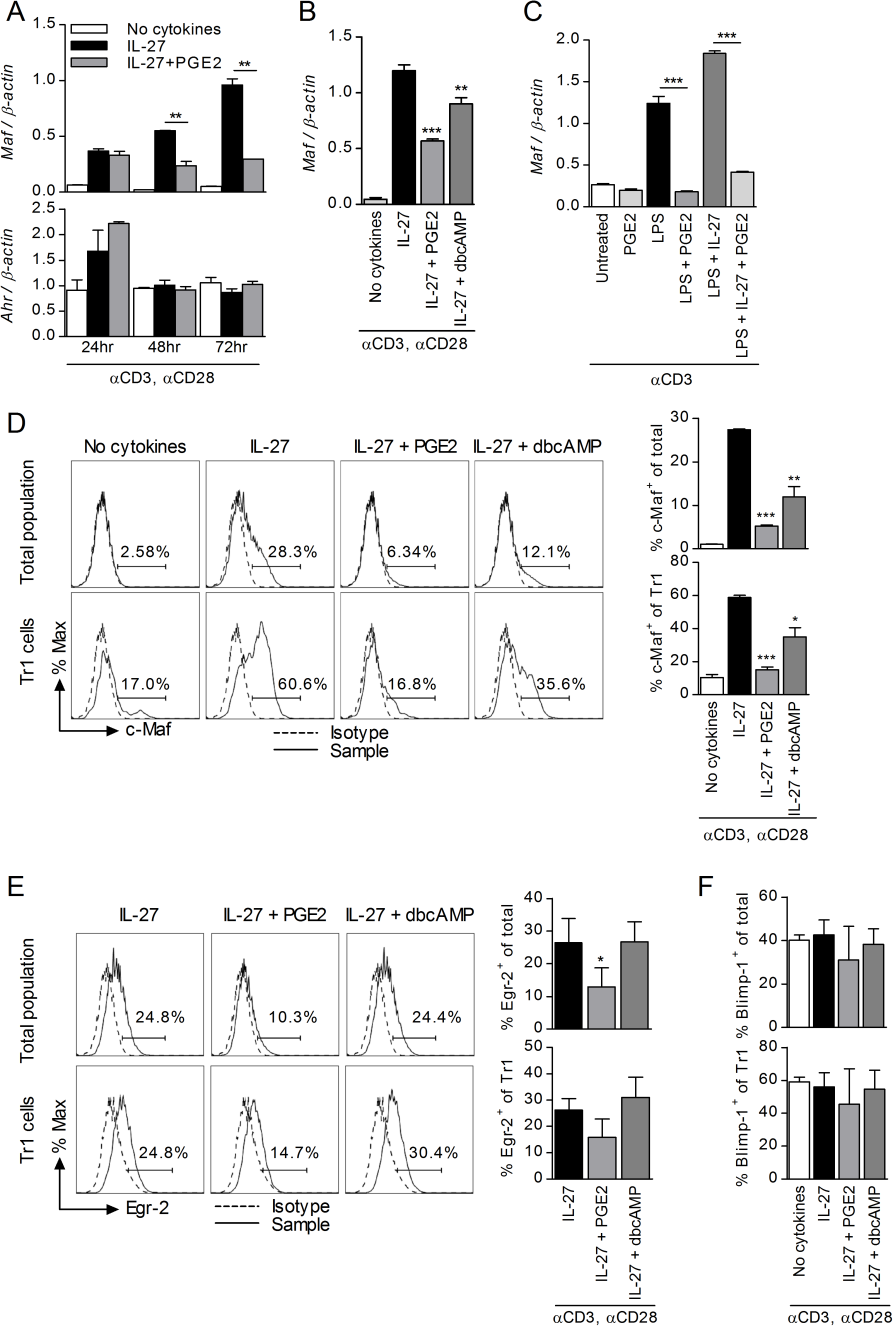
PGE2 inhibits c-Maf in CD4 T cells differentiated in the presence of IL-27. (A) Naïve CD4^+^CD62L^+^ cells were stimulated in the presence of IL-27 and PGE2. RNA was collected at 24, 48 and 72 hr and analyzed by RT-PCR for *Maf* and *Ahr* expression. (B) Naïve CD4^+^CD62L^+^ cells were stimulated in the presence of IL-27 and PGE2 or dbcAMP. Cells were collected on day 3 and analyzed by RT-PCR for *Maf* expression. (C) Splenocytes from *il10*^*gfp*^ mice were stimulated with 3 μg/ml anti-CD3 and 1 μg LPS in the presence of IL-27 and PGE2. RNA was collected on day 3 and *Maf* expression was analyzed by RT-PCR. (D) Naïve CD4^+^CD62L^+^ cells were stimulated in the presence of IL-27 and PGE2 or dbcAMP. Cells were collected on day 3 and analyzed by FACS for intracellular c-Maf within the whole cell population (upper panel) and within CD49b^+^LAG-3^+^ populations (lower panel). Graphical representation of data is on the right. (E) Naïve CD4^+^CD62L^+^ cells were stimulated as in D. Cells were collected on day 3 and analyzed by FACS for intracellular Egr-2 within the whole cell population (upper panel) and within CD49b^+^LAG-3^+^ populations (lower panel). (F) Naïve CD4^+^CD62L^+^ cells were stimulated as in D. Cells were collected on day 3 and analyzed by FACS for intracellular Blimp-1 within the whole cell population and within CD49b^+^LAG-3^+^ populations. Data are representative of two to three independent experiments. Each sample was tested in duplicate and results represent means ± SD. Significance was evaluated by one-way ANOVA and * symbolizes significance of experimental condition versus IL-27 control (B, D, E); *P<0.*05*, **P<0.*01*, ***P<0.*001*.

Previous studies identified c-Maf and AhR as major transcription factors involved in both the differentiation of Tr1 cells and the production of IL-10 by Tr1 cells [14, 21]. To explore the effects of PGE2 on c-Maf and AhR in IL-27 differentiating Tr1 cells, we analyzed first *Maf* and *Ahr* expression by qRT-PCR. Expression of *Maf* increased gradually during Tr1 cell differentiation, while *Ahr* peaked at 24h (Fig 5A). PGE2 inhibited expression of *Maf* at both 48 and 72h, with no effect on *Ahr* expression (Fig 5A). Both PGE2 and dbcAMP inhibited *Maf* expression (Fig 5B), supporting the role of cAMP as a signaling intermediate. The inhibitory effect of PGE2 on *Maf* expression occurred also in unfractionated spleen cell cultures stimulated with LPS and anti-CD3 in the presence and absence of IL-27 (Fig 5C). Next, we confirmed the inhibitory effect of PGE2 and dbcAMP on c-Maf protein levels in differentiating Tr1 cells by using intracellular flow cytometry. Similar to the qRT-PCR data, both PGE2 and dbcAMP significantly reduced intracellular c-Maf in both the total CD4 T cell population and in the CD49b^+^LAG-3^+^ gated Tr1 cells (Fig 5D).

In addition to c-Maf and AhR, Egr-2 and downstream Blimp-1 have been implicated in IL-10 expression in Tr1 cells [22, 25]. Analysis of intracellular Egr-2 in CD4 T cells differentiated in the presence of IL-27 showed a statistically significant reduction by PGE2, but not by dbcAMP, in the total CD4 T population, and a trend towards reduction without reaching statistical significance in the gated Tr1 cells (Fig 5E). The effects on Blimp-1 tested by intracellular flow cytometry did not reach statistical significance and were minimal (Fig 5F). These data indicate that the inhibitory effect of PGE2 on IL-27-induced Tr1 differentiation is associated primarily with substantial reductions in c-Maf expression, with dbcAMP mimicking the effects of PGE2.

### The effect of PGE2 on IL-27-induced Tr1 differentiation is independent of IL-21

**Fig 6:**
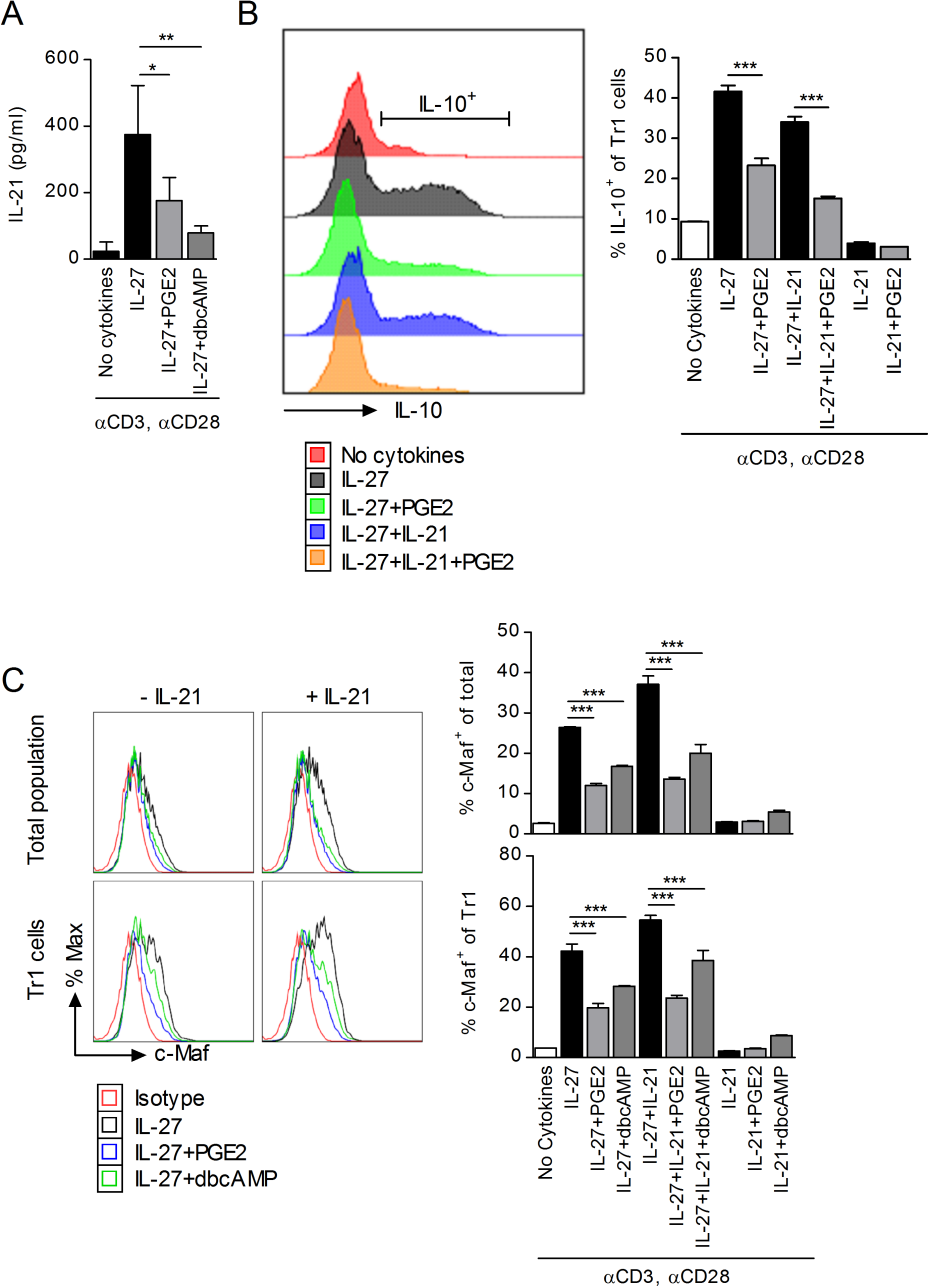
Inhibition of c-Maf by PGE2 is independent of IL-21. (A) Naïve CD4^+^CD62L^+^ cells were stimulated in the presence of IL-27 and PGE2 or dbcAMP. Supernatant was collected on day 3 and analyzed by ELISA for IL-21. Data are cumulative from two independent experiments. (B) Naïve CD4^+^CD62L^+^ cells from *il10*^*gfp*^ mice were stimulated in the presence of IL-27, 100 ng/ml IL-21 and PGE2. Cells were collected on day 3 and analyzed by FACS to determine IL-10 production within CD49b^+^LAG-3^+^ CD4 T cells. Data are representative of two independent experiments. (C) Naïve CD4^+^CD62L^+^ cells from C57BL/6 mice were stimulated as above. Cells were collected on day 3 and analyzed by FACS to determine presence of intracellular c-Maf within CD4 T cells (top) and CD49b^+^LAG-3^+^ CD4 T cells (bottom). Histograms show representative samples, while graphs present data from one of three independent experiments. Each sample was tested in duplicate and results represent means ± SD. Significance was determine by one-way ANOVA; **P<0.*01*, ***P<0.*001*.

Tr1 cells differentiated in the presence of IL-27 secrete substantial amounts of IL-21 that precedes IL-10 secretion [14]. Although IL-21 cannot induce Tr1 differentiation by itself, it plays a crucial role as an autocrine growth factor in the expansion and functional stability of IL-27-induced Tr1 cells primarily by amplifying and stabilizing c-Maf expression [43]. To determine whether PGE2 inhibits the differentiation of Tr1 cells through its effect on IL-21, we first investigated the effect of PGE2 on IL-21 production in IL-27-induced Tr1 cells. Indeed, PGE2 and dbcAMP reduced IL-21 production from Tr1 cells differentiated in the presence of IL-27 (Fig 6A). However, the effect of PGE2 on Tr1 differentiation was not mediated solely through the reduction in IL-21, since we observed decreases in CD49b^+^LAG-3^+^IL-10^+^ T cells both in the presence and absence of exogenous IL-21 (Fig 6B).

IL-27 signaling in CD4 T cells leads to expression of c-Maf and activation of AhR resulting initially in the expression of IL-21R and production of IL-21, which then allows IL-21 to act in an autocrine manner to maintain the high levels of c-Maf expression required for Tr1 differentiation and IL-10 production [14, 43]. In the absence of IL-27, IL-21 cannot initiate c-Maf expression, Tr1 differentiation or IL-10 production. The inhibitory effect of PGE2 on Tr1 differentiation and IL-10 production could be mediated through its inhibitory effect on the initial IL-27 induced expression of c-Maf and/or through the reduction in IL-21 leading to a lack of maintained c-Maf expression. To address the possibility that PGE2 affects c-Maf expression through the reduction in IL-21, we examined c-Maf expression by flow cytometry in CD4 T cells treated with IL-27 in the presence or absence of exogenous IL-21. As expected, treatment with IL-21 in the absence of IL-27 did not result in IL-10 or c-Maf expression (Fig 6B and 6C). In addition, PGE2 was able to inhibit IL-10^+^ Tr1 cell differentiation and c-Maf expression in IL-27 treated CD4 T cells, both in the absence and presence of exogenous IL-21 (Fig 6B and 6C). These results indicate that, although PGE2 reduces IL-21 production, the inhibition of c-Maf expression induced by IL-27 represents the major mechanism for the inhibitory effect of PGE2 on Tr1 differentiation.

### The effects of PGE2 on Tr1 differentiation and c-Maf expression do not depend on STAT1 or STAT3 activation

**Fig 7:**
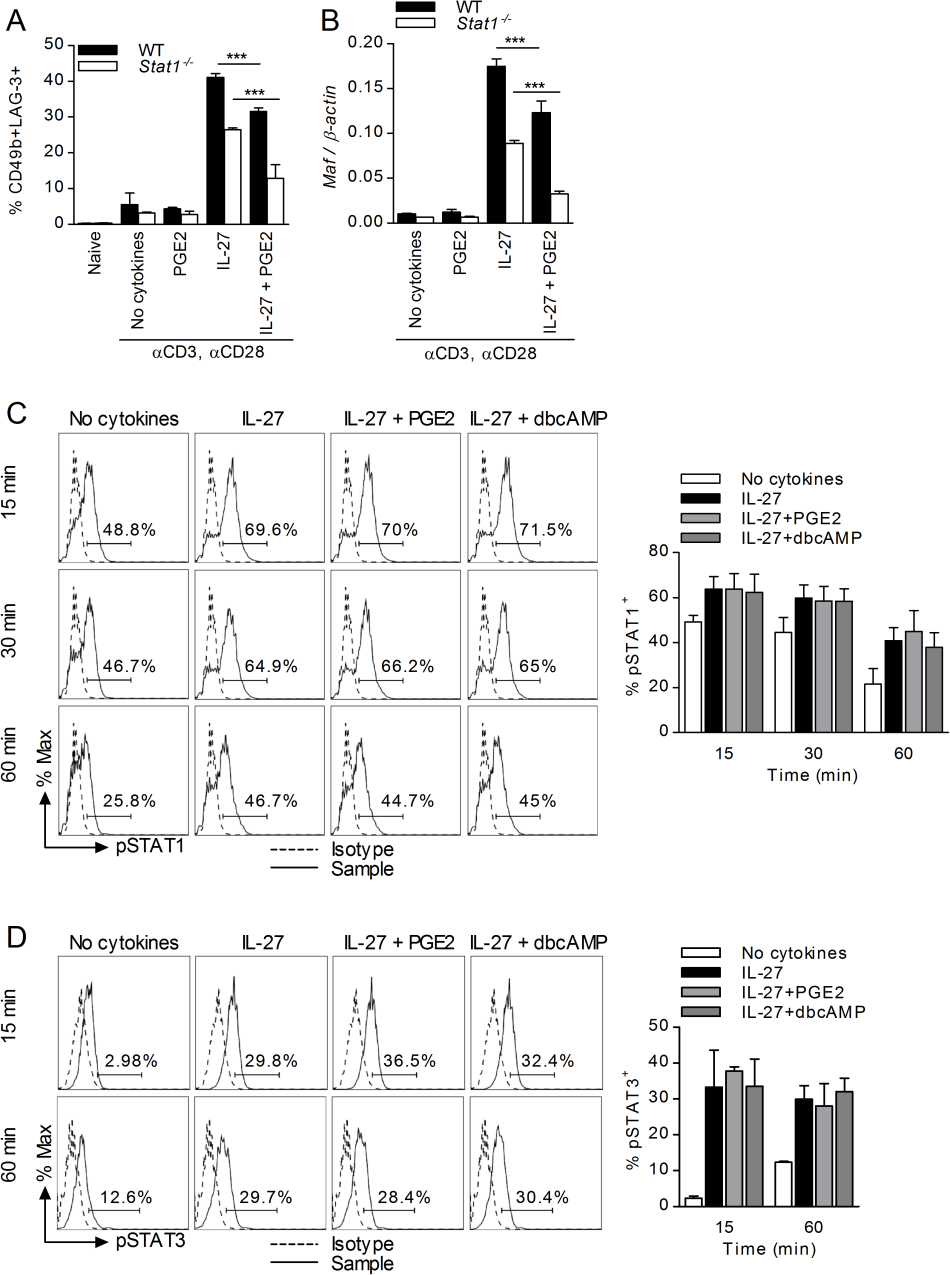
Inhibition of Tr1 differentiation by PGE2 is independent of STAT1/3. Naïve CD4^+^CD62L^+^ cells from wildtype 129S6 (WT) and *Stat1*^*-/- *^mice were stimulated in the presence of IL-27 and PGE2. (A) Samples were collected on day 3 and cells were analyzed for Tr1 markers. (B) Cells were subjected to RNA extraction and subsequent analysis by RT-PCR for *Maf* expression. (C) Naïve CD4^+^CD62L^+^ cells from C57BL/6 mice were stimulated in the presence of IL-27, PGE2 and 10^-4^M dbcAMP. Cells were collected 15, 30 60 minutes later and analyzed by FACS for intracellular phospho-STAT1 (Tyr701). (D) Naïve CD4^+^CD62L^+^ cells from C57BL/6 mice were stimulated as described in (C) for 15 and 60 minutes. Cells were analyzed by FACS for intracellular phospho-STAT3 (Tyr705). Data are cumulative from three independent experiments. Each sample was tested in duplicate and results represent means ± SD. Significance was determined using one-way ANOVA; ***P<0.*001*.

In T cells, engagement of the IL-27 receptor leads to activation of STAT1 and STAT3 signaling, resulting in inhibition of Th17 and promotion of Tr1 differentiation [13, 18, 54]. In contrast to Th17 differentiation which is inhibited by STAT1, Tr1 differentiation and IL-10 production are promoted by both STAT1 and STAT3 activation [13, 14, 18]. Therefore, we investigated whether the inhibitory effect of PGE2 was mediated through effects on STAT1 or STAT3 activation. To determine the role of STAT1, we assessed the effect of PGE2 on Tr1 differentiation of wildtype and *Stat1*^*-/-*^ CD4 T cells. Although IL-27 treatment of *Stat1*^*-/-*^ T cells resulted in fewer CD49b^+^LAG-3^+^ Tr1 cells (~40% reduction), PGE2 inhibited Tr1 differentiation at the same level as in wildtype CD4 T cells (Fig 7A). Similarly, overall expression of *Maf* was reduced in IL-27 treated *Stat1*^*-/-*^ T cells, but PGE2 maintained its inhibitory activity (Fig 7B). To examine whether PGE2 affected activation of STAT1, we determined the levels of STAT1 phosphorylation (Tyr701) by flow cytometry in stimulated CD4 T cells treated with IL-27 in the presence or absence of PGE2 or dbcAMP. IL-27-induced phosphorylation of STAT1 was unaffected by either PGE2 or dbcAMP (Fig 7C). These results confirmed the role of STAT1 in Tr1 differentiation but excluded STAT1 as a mediator for the inhibitory effect of PGE2. To examine whether PGE2 affected STAT3 activation, we treated naïve CD4 T cells with IL-27 in the presence or absence of PGE2 or dbcAMP, and determined the levels of STAT3 phosphorylation (Tyr705) by flow cytometry. While IL-27 increased STAT3 phosphorylation, neither PGE2 nor dbcAMP affected IL-27-induced STAT3 phosphorylation (Fig 7D), suggesting that the inhibitory effect of PGE2 was not mediated through a reduction in STAT3 activation.

### The in vivo effect of PGE2 on Tr1 cells

**Fig 8:**
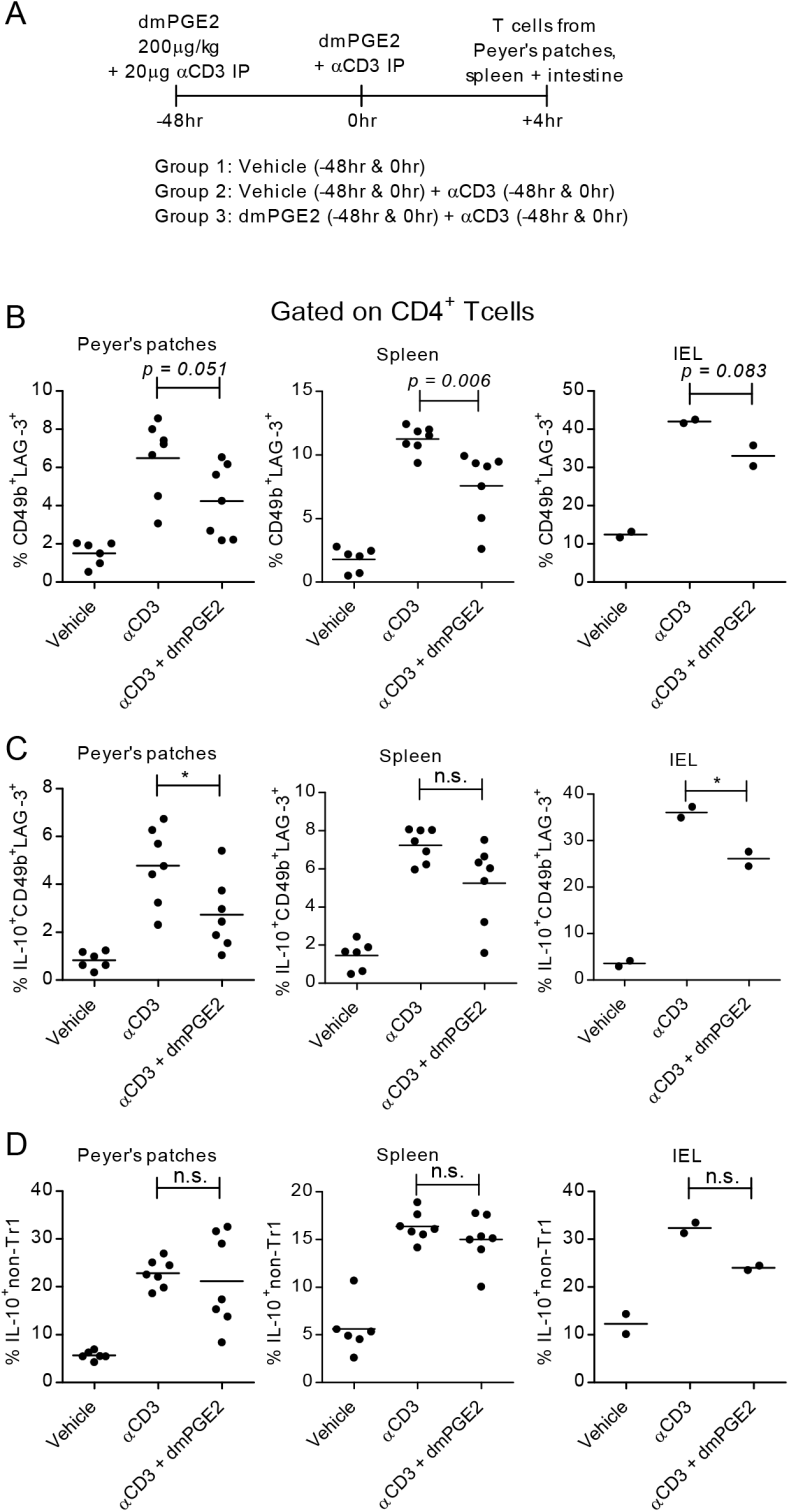
PGE2 inhibits Tr1 differentiation in vivo. (A) Schematic of timeline and experimental groups. *Il10*^*gfp*^ mice were injected i.p. with vehicle, 20 μg/ml anti-CD3 or anti-CD3 and 4μg dmPGE2 at 0hr and 48hr. Peyer’s patches, spleen, and intraepithelial lymphocyte populations were collected. (B) CD4^+^ populations were analyzed by FACS for Tr1 cell populations (CD49b^+^LAG-3^+^). (C-D) CD4^+^ populations were analyzed by FACS for IL-10 expression in both Tr1 populations and non-Tr1 populations (including CD49b^+^LAG-3^-^, CD49b^-^LAG-3^+^ and CD49b^-^LAG-3^-^ populations). Data are cumulative of two independent experiments. Significance was evaluated by unpaired t-test.

To determine whether treatment with PGE2 affects Tr1 generation/amplification in vivo, we used a model of anti-CD3-mediated transient intestinal inflammation [55]. *Il10*^*gfp*^ mice were inoculated i.p. with anti-CD3 and the stable PGE2 analog dimethyl PGE2 (dmPGE2), and cells from Peyer’s patches, spleen, and intraepithelial lymphocytes (IEL) from the small intestine were obtained 4h after the last inoculation (Fig 8A). The percentage of CD4^+^CD49b^+^LAG-3^+^ Tr1 cells, IL-10^+^ Tr1, and IL-10^+^ non-Tr1 CD4 populations were determined by flow cytometry. Both CD4^+^CD49b^+^LAG-3^+^ Tr1 cells and the percentage of IL-10^+^ Tr1 and non-Tr1 CD4^+^ cells increased following anti-CD3 inoculation in all tissues examined. Administration of dmPGE2 led to a reduction in the percentage of CD4^+^CD49b^+^LAG-3^+^ cells, IL-10^+^CD49b^+^LAG-3^+^ cells, and IL-10^+^ non-Tr1 CD4^+^ T cells, reaching statistical significance in spleen and Peyer’s patches for CD4^+^CD49b^+^LAG-3^+^ cells and in IELs for IL-10^+^CD49b^+^LAG-3^+^ (Fig 8B–8D).

## Discussion

PGE2, a major prostanoid abundant at inflammation sites, has multiple effects on the immune response primarily through EP2 and EP4 receptors expressed on immune cells [46]. In terms of CD4 T cell differentiation, recent studies using EP2 and EP4-deficient mice and specific EP2/EP4 antagonists concluded that PGE2 induced Th1 differentiation and facilitated Th17 expansion in models of immune inflammation [48]. In agreement with the in vivo proinflammatory role of PGE2, here we report for the first time that PGE2 inhibits the IL-27-induced differentiation of regulatory Tr1 cells. The inhibitory effect of PGE2 on the differentiation and IL-10 production by CD4^+^CD49b^+^LAG-3^+^ Tr1 cells is mediated by EP4 and cAMP leading to a significant reduction in c-Maf expression, without affecting STAT1/STAT3 activation. The effect of PGE2 on CD4^+^CD49b^+^LAG-3^+^ Tr1 differentiation is not associated with either induction of Foxp3 or IL-17 production, suggesting a lack of transdifferentiation into Foxp3^+^ Treg or effector Th17 cells. Whether other cAMP signaling prostaglandins such as PGD2 and PGI2 affect Tr1 differentiation remains to be determined. In terms of effects on Foxp3+ regulatory T cells, cAMP signaling by PGD2 and PGI2 had opposite effects, with PGD2 promoting inducible Treg indirectly through its effects on cDC, and PGI2 directly inhibiting human Foxp3 Treg differentiation [56, 57]. The effect of PGE2 on Tr1 differentiation is novel. Previous studies related to PGE2 and CD4 T cell differentiation focused primarily on effector T cells. Although PGE2 was reported initially to inhibit Th1 differentiation, this was apparently due to the PGE2-induced suppression of IL-12 production in APCs [29, 58]. More recently, PGE2 was shown to promote Th1 differentiation in vivo and in vitro through upregulation of IL-12Rβ2 and IFNγ-R1 on CD4 T cells [33, 47]. PGE2 was also reported to promote differentiation of Th17 cells through upregulation of IL-1R and IL-23R, and Th17 expansion by altering the IL-23/IL-12 balance in favor of IL-23 in cDC [34, 45].

The role of PGE2 as a pro-inflammatory agent was confirmed in vivo in models of contact hypersensitivity, IBD, RA and EAE, with EP4 identified as the major mediator leading to Th1/Th17 differentiation [32, 38, 47, 59–61]. The present study showing that PGE2 inhibits both Tr1 differentiation and IL-10 production in Tr1 cells brings an additional mechanistic perspective to the proinflammatory role of PGE2.

CD4 Tr1 cells, characterized by co-expression of CD49b, LAG-3 and CD226, high levels of IL-10 production, and lack of constitutive Foxp3 and CD25 expression, play a major role in tolerance and immune homeostasis [62, 63]. Tr1-mediated immune suppression occurs through multiple mechanisms including secretion of high levels of IL-10 and TGFβ, generation of extracellular adenosine, direct killing of APCs through granzyme B/perforin release, and PD-1/PD-L and CTLA-4-mediated inhibition of the stimulatory capacity of APCs [63, 64]. The generation of Tr1 cells has been studied extensively [63, 65]. In addition to IL-27, which was identified as the crucial factor in inducing Tr1 differentiation [18, 19], both IL-21 and IL-10 were reported to act as autocrine factors in Tr1 amplification and maintenance of functional stability [43, 66].

Initially, IL-27 was described as promoting Th1 differentiation [67, 68]. However, in contrast to expectations, mice deficient in IL-27R were able to mount adequate Th1 responses against intracellular pathogens [69], but succumbed due to uncontrolled severe tissue inflammation associated with exaggerated T cell responses and enhanced TNF and IFNγ production [70]. These observations led to the conclusion that IL-27 acted primarily by limiting the response of effector T cells and by maintaining and/or reestablishing immune homeostasis [71, 72]. Subsequent studies demonstrated the role of IL-27 in inducing Tr1 cells in vitro and in vivo, and revealed its therapeutic potential [11–13, 15, 17].

In contrast to factors that positively affect Tr1 differentiation, much less is known about the suppression of Tr1 development and/or function. It is conceivable that factors present in acute inflammatory conditions, and possibly in chronic inflammation as well, might have a negative impact on Tr1 development and function. A recent study reported that extracellular ATP and hypoxia, both increased at inflammatory sites, inhibit Tr1 differentiation through AhR inactivation [26]. Along these lines, most prostanoids and especially PGE2, found at high concentrations at inflammatory sites, could impact the generation and function of Tr1 cells either indirectly through effects on IL-27 production or directly by acting on the differentiating Tr1 cells. We reported recently that PGE2 inhibits the expression and production of IL-27 from TLR-stimulated cDC by reducing IRF1 expression and binding to the *Il27p28* promoter [41]. Here, we report that PGE2 can also directly affect IL-27-induced Tr1 differentiation by reducing the expression of the essential transcription factor c-Maf in Tr1 differentiating CD4 T cells.

c-Maf is a critical factor in Tr1 differentiation and IL-10 expression, acting in conjunction with AhR for the transactivation of both *Il10* and *Il21* transcription [14, 21]. The essential role of c-Maf and AhR was also demonstrated in vivo for the IL-27-mediated induction of Tr1 cells [17]. In addition to c-Maf and AhR, IL-10 production in Tr1 cells is also dependent on the Egr-2→Blimp-1 pathway controlled primarily through STAT3 activation [22]. We found that PGE2 significantly reduces c-Maf expression at both mRNA and protein level during Tr1 differentiation, with no effect on AhR and minimal effects on Egr-2→Blimp-1 expression. Kuchroo and colleagues proposed that Tr1 differentiation occurs in two phases, *i*.*e*. the initiation phase induced by IL-27, and the IL-21/IL-10-dependent Tr1 amplification/stabilization [14]. c-Maf was shown to play a major role in both the initiation and amplification phase. In the initiation phase, signaling through IL-27R induces expression of c-Maf, IL-21R and ICOS, then c-Maf transactivates the *Il21* and *Il10* promoters. In the amplification phase, IL-21 acts in an autologous manner activating and maintaining high levels of c-Maf expression. Our finding that PGE2 inhibits c-Maf expression during Tr1 differentiation is in agreement with the observed inhibitory effect on the production of IL-21 in the initiation phase. The inhibitory effect of PGE2 on Tr1 differentiation could be entirely mediated through the inhibition of IL-21 expression. However, we found that PGE2 inhibits the differentiation of CD49b^+^LAG-3^+^IL10^+^ Tr1 and reduces c-Maf levels in Tr1 cells even in the presence of exogenous IL-21. These observations suggest that the inhibitory effect of PGE2 is mediated primarily through reduction in c-Maf expression, affecting both Tr1 induction and amplification/stabilization.

Although human and murine CD4 T cells express both EP4 and EP2 receptors, loss of function experiments and use of selective receptor antagonists identified EP4 as a major mediator of PGE2-induced Th1/Th17 differentiation and expansion in models of immune inflammation [48]. We found that the inhibitory effect of PGE2 on Tr1 cells is also mediated through EP4. EP4 signals through activation of adenylate cyclase and PI3K. The effects of PGE2 on IL-21 production, Tr1 differentiation and c-Maf expression were mimicked by dbcAMP, strongly suggesting that the effect of PGE2 is mediated through cAMP. Similar conclusions were previously reached for the effects of PGE2 on Th1 and Th17 differentiation [48]. However, in contrast to previous reports which identified PKA or EPAC as downstream mediators, we found that neither one was involved in the inhibitory effect of PGE2 on Tr1 differentiation. We eliminated STAT1 and STAT3, known intermediates in IL-27-mediated Tr1 differentiation [13, 73], as possible targets for the inhibitory effect of PGE2 and dbcAMP. The link between cAMP induction and inhibition of c-Maf expression remains unidentified at the present time. Future transcriptome studies are required to elucidate the signals involved in the PGE2→cAMP→c-Maf pathway.

Fate mapping experiments underlined the plasticity of both Th17 and Foxp3^+^ Treg, and a recent study reported on the transdifferentiation of Th17 into Tr1 cells during resolution of inflammation [44, 74, 75]. PGE2 was previously reported to promote Th17 expansion and to induce Foxp3 expression and regulatory function in human T cells [32, 39]. Therefore, we took into consideration the fact that PGE2 might switch Tr1 differentiation towards either Foxp3 expressing- or IL-17-producing T cells. This was not the case. However, the questions whether already differentiated Tr1 cells could be induced to transdifferentiate into effector T cells in an inflammatory microenvironment, and whether PGE2 could contribute to this transdifferentiation, remain to be addressed.

## Supporting information

S1 Fig Differentiation of Tr1 and Treg cells. Naïve CD4^+^CD62L^+^ cells were stimulated with plate-bound anti-CD3 (3 μg/ml) and soluble anti-CD28 (1 μg/ml) in the presence of 5 ng/ml TGF-β1 or 50 ng/ml IL-27 for three days. Cells were collected on day 3 and analyzed by flow for intracellular FOXP3 or Tr1 markers CD49b & LAG-3. Each sample was tested in duplicate and results represent means ± SD. Significance was determined using one-way ANOVA; ***P<0.*001*.
(TIF)
