# Supplementary figures and images for: Prostaglandin E2 inhibits Tr1 cell differentiation through suppression of c-Maf
Source: PLoS One. 2017 Jun 12;12(6):e0179184. doi: 10.1371/journal.pone.0179184 (PMC5467903; doi:10.1371/journal.pone.0179184)

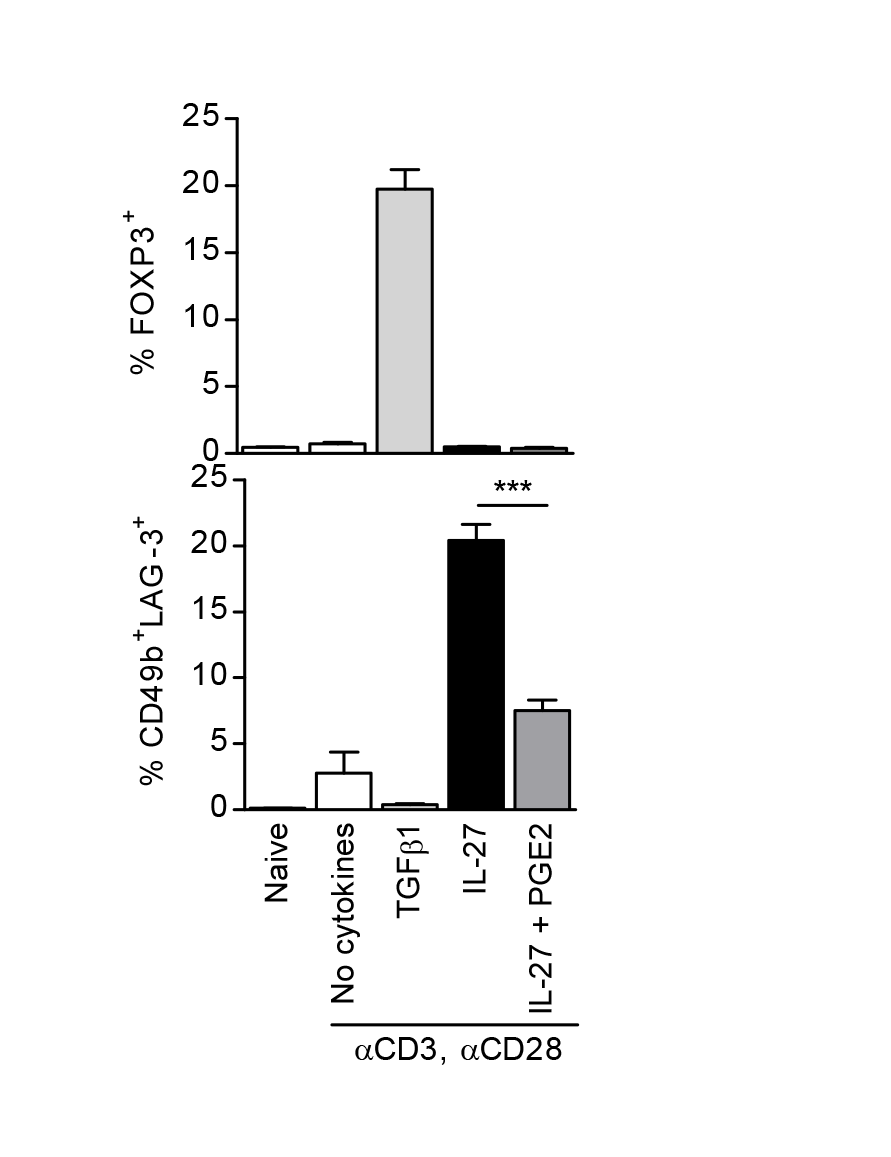

Supplement: S1 Fig — Naïve CD4+CD62L+ cells were stimulated with plate-bound anti-CD3 (3 μg/ml) and soluble anti-CD28 (1 μg/ml) in the presence of 5 ng/ml TGF-β1 or 50 ng/ml IL-27 for three days. Cells were collected on day 3 and analyzed by flow for intracellular FOXP3 or Tr1 markers CD49b & LAG-3. Each sample was tested in duplicate and results represent means ± SD. Significance was determined using one-way ANOVA; ***P<0.001. (TIF) [file pone.0179184.s001.tif]
